# Simulation and Experimental Study on Reverse Helical Milling with the Gradual-Removal Reverse Edge Milling Cutter under Ultrasonic-Assisted Condition

**DOI:** 10.3390/ma15031117

**Published:** 2022-01-31

**Authors:** Kemeng Ren, Guangyue Wang

**Affiliations:** Key Laboratory of Advanced Manufacturing and Intelligent Technology, Ministry of Education, Harbin University of Science and Technology, Harbin 150080, China; renkemeng050@163.com

**Keywords:** carbon fiber-reinforced plastics, bi-direction helical milling, cutting tool design, finite element method, hole quality

## Abstract

As a new machining method, ultrasonic-assisted bi-direction helical milling has obvious advantages in making holes on carbon fiber-reinforced plastics (CFRP). However, cutting edges of the flat-bottomed milling cutter are easy to wear, which may cause severe defects such as burrs and tears in the outlet of the hole. In order to improve the hole-making quality of CFRP, the gradual-removal reverse edge milling cutter was proposed and designed. The finite method models of reverse helical milling CFRP with the flat-bottomed reverse edge milling cutter and the gradual-removal reverse edge milling cutter under an ultrasonic vibration were established, and the comparative cutting experiments of the two cutters were carried out. By comparing the cutting performance of the two milling cutters under the condition of ultrasonic vibration assistance, the cutting mechanism of improving the hole wall quality by the gradual-removal reverse edge milling cutter was studied. The results showed that when the reverse cumulative cutting depth reached about 60 mm, compared with the flat-bottomed reverse edge milling cutter, the gradual-removal reverse edge milling cutter transferred part of the cutting task of the peripheral edge to the end edge, and the wear of the reverse peripheral edges which directly affects the hole quality was effectively alleviated. This mechanism made the cutting state of the peripheral edge dominated by shear failure, which led to the significant improvement of the quality at the outlet of the hole.

## 1. Introduction

Carbon fiber-reinforced plastics (CFRP) is widely used in advanced manufacturing fields, such as aviation and aerospace, because of its low density, high strength and excellent mechanical properties [1,2]. CFRP is mostly used as key structural parts in aircrafts, and a large number of high-precision assembly connecting holes are often required to be machined in the assembly process [3]. The helical milling method is gradually becoming widely used in CFRP high-precision hole making because of its advantages, such as low cutting temperature, fast heat dissipation, large chip removal space, small axial force, short machining time, and good hole quality [4]. Nevertheless, the cutting edges of the CFRP helical milling cutter still suffer from rapid wear, which easily leads to defects such as burrs and tears at the outlet of the hole [5,6].

**Figure 1:**
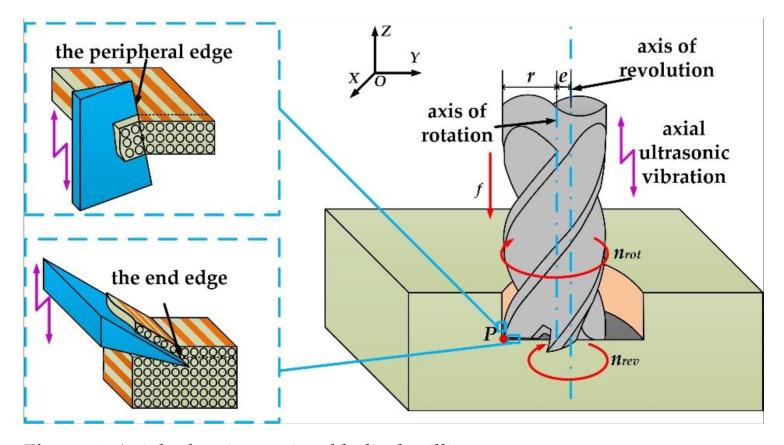
Axial vibration assisted helical milling.

Different from the traditional drilling method, the movement form of the helical milling tool is more complex, which consists of two parts. The first one is a spiral feed movement along the axis of the hole, and the other one is the rotational motion around the tool axis. Ultrasonic vibration assisted machining is to apply a high-frequency vibration higher than 15 KHz to the tool or workpiece, thereby forming a high-frequency composite cutting movement of the tool or workpiece, which is a typical composite machining technology. Figure 1 reflects the influence of axial ultrasonic-assisted vibration on the motion forms of the end edge and the peripheral edge of the tool in the helical milling process. Axial vibrations can change the motion form of the tool, thereby affecting the material removal behavior.

In order to clarify the kinematic mechanism of helical milling, the trajectory equation of tool tip P, changing with time under ultrasonic-assisted conditions, was established. By taking the hole center as the intersection point of the X axis and the Y axis, and the hole axis as the Z axis, the OXYZ coordinate system is established. The trajectory equation is shown in Equation (1).

(1) $$\left\{\begin{array}{l} x=e\cos(\frac{2\pi n_{rev}t}{60})+r\cos(\frac{2\pi n_{rot}t}{60})\\ y=e\sin(\frac{2\pi n_{rev}t}{60})+r\sin(\frac{2\pi n_{rot}t}{60})\\ z=h-ft+A\sin(2\pi f_{U}t) \end{array}\right.$$

where *x*, *y*, *z* are the three-dimensional coordinates of the tip point, respectively, *e* is eccentricity, *n_rev_* and *n_rot_* are the tool rotation speed and rotation speed, respectively, *r* is milling cutter radius, *h* is the thickness of the processed workpiece, *t* is cutting time, *f* is the axial feed rate, *A* is the axial vibration amplitude, and *f_u_* is the axial vibration frequency.

In terms of CFRP machining in a helical milling way, Sadek et al. [7] carried out comparative experimental research on CFRP helical milling and traditional drilling, and the results showed that, compared with conventional drilling, the cutting temperature and axial force of helical milling holes were lower and the quality of holes were significantly improved. Wang et al. [8] put forward a comparative experimental scheme for helical milling and drilling of CFRP with the same feed speed, and the results showed that the lower cutting temperature was the main reason for the higher quality of helical milling holes. Amini et al. [9] evaluated the effects of cutting speed, feed rate and other cutting parameters on the surface quality of the hole by using the full factor method, and the results showed that the cutting speed was the key factor determining the diameter, roundness and cylindricity of the hole. Brinksmeier et al. [10] derived the cutting ratio of the front and peripheral edges of the flat-bottomed milling cutter, and the results showed that the cutting ratio of the two parts was only affected by the ratio of eccentricity and hole radius. HAN et al. [11] carried out a comparative study on the cutting performance of the TiB2 coated milling cutter and the diamond coated milling cutter, and the results showed that the diamond coated cutter had obvious advantages in both cutter life and cutting force. Chen et al. [12] proposed and designed a new type of chip-splitting helical milling cutter, and it was found that the cutter could effectively reduce the chip size and prolong the cutter life through experimental verification. Yang et al. [13] proposed the forward and reverse helical milling strategy and carried out the experimental verification, and the results showed that when this machining method was used to reversely cut a CFRP workpiece, the material at the export was supported by its own unprocessed material, which effectively inhibited the delamination at the exit. Ishida et al. [14] proposed a composite helical milling technology combining ultrasonic vibration and cryogenic cutter cooling, and the results showed that the processing method could effectively reduce the axial force and significantly improve the quality of the hole. Chen et al. [15] carried out a comparative experimental study of conventional and ultrasonic assisted bi-direction helical milling of CFRP, and found that the ultrasonic assisted helical milling method changed the material removal mode and inhibited the processing damage caused by insufficient support at the exit of the fiber bundle. Liu et al. [16] studied the effects of axial vibration and torsional vibration on the cutting speed and acceleration, and found that the ultrasonic vibration assisted machining method could better remove carbon fibers with different arrangement directions.

Table 1 briefly summarizes the main research objects and main parameters of hole processing of the above researches. The above researches show that, compared with the traditional drilling, the cutting temperature and cutting force of the helical milling method are smaller, and the surface quality of the hole is significantly improved. In order to further extend the tool life and improve the quality of helical milling holes, many experimental studies have been carried out on optimizing cutting parameters, improving tool structure, and introducing forward and reverse machining methods and ultrasonic assisted machining methods. However, the problem of quality decline at the outlet of holes caused by tool wear is still difficult to avoid. Therefore, how to improve the tool structure, prolong the tool life and realize high quality drilling has become the key to the promotion of helical milling.

In recent years, many researchers have established a series of finite element cutting simulation models to further study the cutting mechanism. Xu et al. [17] established the oblique cutting and continuous cutting model to study the mechanism of drilling CFRP. He et al. [18] introduced a subroutine to describe the constitutive equation of CFRP material, and established the three-dimensional macroscopic mechanical model of CFRP drilling. Phadnis et al. [19] established the macroscopic drilling mechanics model of CFRP, and in this model, anisotropic CFRP materials staggered along four directions to simulate the fiber arrangement of quasi-isotropic plates. Ji et al. [20] studied the influence of spindle speed and axial feed speed on cutting force through the helical milling simulation model of Ti6Al-4V alloy. Zhang et al. [21] used a cohesive element method to establish the viscous interface between fiber layers, and predicted the delamination initiation in rotary ultrasonic machining. Abena et al. [22] introduced the SPH method to simulate the finite element model of orthogonal cutting CFRP at a micro scale in order to solve the model distortion problem caused by element deletion, and the model could better predict the chip formation process. The finite element method has been proved to provide an intuitive and powerful basis for in-depth study of the cutting mechanism. However, the current research is still focused on demonstrating the accuracy of the simulation model, and there are still few cases of practical application of the finite element method in the structural design of CFRP helical milling cutters.

In summary, the ultrasonic assisted forward and reverse helical milling method can effectively inhibit the damage at the exit of CFRP holes, but the reverse edge of the existing flat-bottomed milling cutter wears fast, and the quality at the outlet of the hole decreases rapidly with the bluntness of the edge. In order to solve this problem, this paper improved the reverse cutting edge of the cutter. A gradual-removal reverse edge milling cutter was proposed and designed which can reduce the cutting ratio of the peripheral edge. Through the establishment of a three-dimensional cutting simulation model and undeformed chip model, the cutting performance and cutter life of the reverse edge milling cutter were analyzed. Combined with experimental verification, the cutting mechanism and wear mechanism of the gradual-removal reverse edge milling cutter were studied in detail.

## 2. Establishment of Finite Element Model

Since the explicit dynamics method is easy to converge and has good stability, it can effectively avoid the divergence problem in the cutting simulation. The Lagrange method focuses on studying the dynamic change law of each element particle with time in the simulation model. In order to obtain the cutting force data of the tool and the stress distribution of the workpiece in the process of axial ultrasonic assisted helical milling, this paper selects the explicit dynamics and Lagrange method module in Abaqus 2019 (SIMULIA, Providence, RI, USA) and simulates the ultrasonic assisted helical milling movement by coupling the constrained element, introducing the velocity displacement function, and modifying the input file. A three-dimensional cutting simulation model of a flat bottom reverse edge milling cutter and involute reverse edge milling cutter is established to remove the material at the exit.

### 2.1. Failure Mode of CFRP

The workpiece material is a CFRP/T700/30° unidirectional composite plate, which is a homogeneous anisotropic material with elastic failure behavior. In order to simulate the failure mode of fibers, the Hashin failure criterion was used to describe the material constitutive, and the three-dimensional elastic constitutive was constructed through the custom subroutine (VUMAT). The material properties are shown in Table 2.

The Hashin criterion contains four different failure modes. When any of the following damage criterion values reach 1, the material will fail:

Fiber tensile (*σ*_11_ ≥ 0):

(2) $$F_{ft}={\left(\frac{\sigma_{11}}{\sigma_{11}^{T}}\right)}^{2}+{\left(\frac{\tau_{12}}{S_{12}}\right)}^{2}+{\left(\frac{\tau_{13}}{S_{13}}\right)}^{2}$$

Fiber compression (σ_11_ < 0):

(3) $$F_{fc}={\left(\frac{\sigma_{11}}{\sigma_{11}^{C}}\right)}^{2}$$

Fiber compression (*σ*_22_ + *σ*_33_ ≥ 0):

(4) $$F_{mt}={\left(\frac{1}{\sigma_{22}^{T}}\right)}^{2}{\left(\sigma_{22}+\sigma_{33}\right)}^{2}+{\left(\frac{1}{S_{23}}\right)}^{2}\left(\tau_{12}{}^{2}-\sigma_{22}\sigma_{33}\right)+{\left(\frac{1}{S_{12}}\right)}^{2}\left(\tau_{12}{}^{2}+\tau_{13}{}^{2}\right)$$

Compression failure perpendicular to fiber direction (*σ*_22_ + *σ*_33_ < 0):

(5) $$F_{mc}=\frac{1}{\sigma_{22}^{C}}\left[{\left(\frac{\sigma_{22}^{C}}{2S_{23}}\right)}^{2}-1\right]\left(\sigma_{22}+\sigma_{33}\right)+{\left(\frac{1}{2S_{23}}\right)}^{2}{\left(\sigma_{22}+\sigma_{33}\right)}^{2}+{\left(\frac{1}{S_{23}}\right)}^{2}\left(\tau_{23}{}^{2}-\sigma_{22}\sigma_{33}\right)+{\left(\frac{1}{S_{12}}\right)}^{2}\left(\tau_{12}{}^{2}+\tau_{13}{}^{2}\right)$$

*F_ft_*, *F_fc_*, *F_mt_*, *F_mc_* are, respectively, the damage values of four fiber failure modes. When the damage value is equal to 1, the unit fails. *σ^T^*_11_, *σ^C^*_11_, *σ^T^*_22_, *σ^C^*_22_ are, respectively, tensile failure stress in the fiber direction, compressive failure stress in the fiber direction, failure stress perpendicular to the fiber direction and failure stress perpendicular to the compression direction. *S*_12_, *S*_13_, *S*_23_ are the shear failure stresses of three planes, and *σ*_11_, *σ*_22_*, σ*_33_ and *τ*_12_, *τ*_13_, *τ*_23_ are the components of normal stress and shear stress in 3 directions of integral points.

### 2.2. Geometric Models, Materials and Boundary Conditions

**Figure 2:**
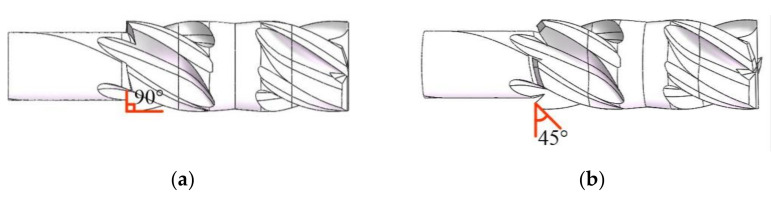
Structures of two milling cutters. (**a**) The flat-bottomed reverse edge milling cutter; (**b**) the gradual-removal reverse edge milling cutter.

The two kinds of bi-direction milling cutters are both composed of a forward cutting zone and reverse cutting zone. The difference is that the reverse edges of the flat-bottomed reverse edge milling cutter are straight, while the reverse edges of the gradual-removal cutting reverse end edge are polygonal-shape cutting edges. In the front view of the milling cutter, the angle between the direction of the cutting edge and the original end edge is 45°, forming a gradual cutting structure. As shown in Figure 2, the diameter of the two milling cutters is 8 mm, the number of teeth is 4, the front angle is 10°, the clearance angle is 8°, and the helix angle of the cutting edge is 35°. The cutter material properties are shown in Table 3.

**Figure 3:**
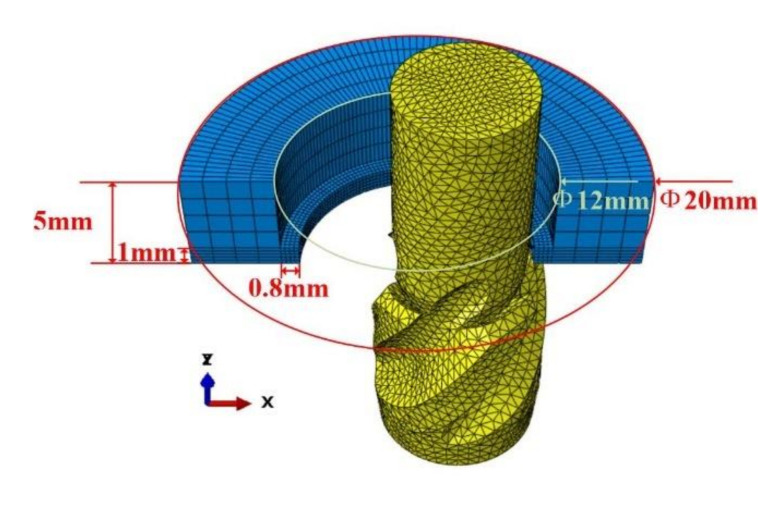
FEM simulation model.

**Figure 4:**
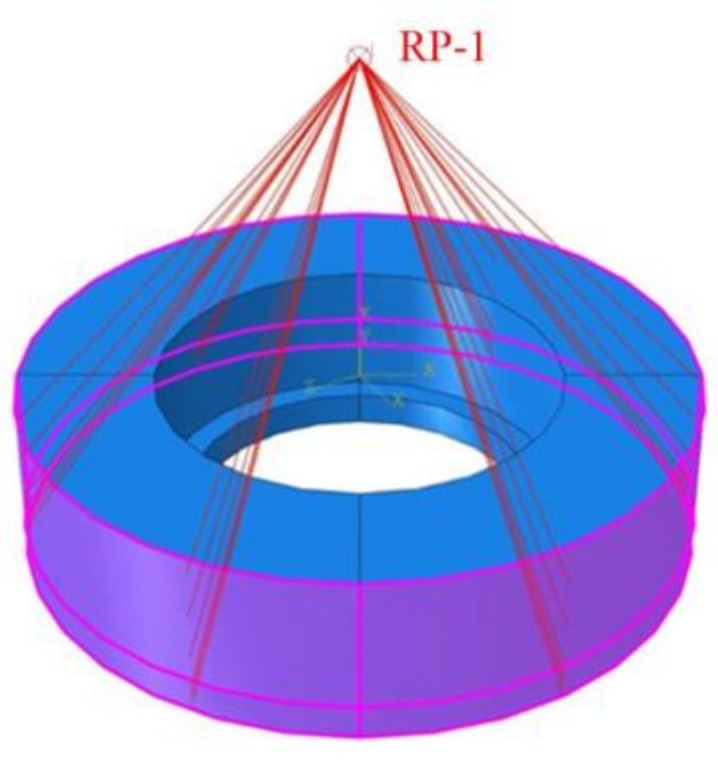
Couple the outer wall.

In order to simplify the model, the finite element simulation model only retained the reverse cutting area of the cutter and the remaining part of the material after forward machining. The mesh size of the cutter part is 0.5 mm, and the element property is the three-dimensional stress C3D4 element (four-node linear tetrahedron element). The workpiece layout needs to be divided into the inner part of the hole and the outer part of the hole. The mesh size of the inner part of the hole is 0.2 mm, the outer distribution of the hole is 1 mm, and the element attribute is the three-dimensional stress C3D8R element (eight-node linear hexahedron element). The advantage of partition block meshing is that it can improve the operation speed on the premise of ensuring the accuracy of the model. In the simulation, the lower surface of the workpiece was located in the XOY plane, the cutter spindle was parallel to the Z axis, the handle was located in the upper part, and the cutting part was located below. The distance between the cutter and the center of the hole in the XY plane is equal to the eccentricity. The finite element simulation model after meshing and assembling is shown in Figure 3. In order to avoid the cutter passing through the workpiece after the failure of the external element of the workpiece, the model modifies the internal file and successfully makes the surface element set cover the internal element. In addition, the outer wall of the workpiece was coupled to the reference point RP-1, and the reference point was located on the central axis of the machining hole, as shown in Figure 4. In order to simulate that the workpiece was fixed on the fixture, the hole wall coupling reference point RP-1 was completely fixed in the initial boundary conditions, and the coupling support reaction output force by RP-1 is the cutting force of the cutter.

The motion form of the workpiece is adjusted by the boundary load. Equations (6) and (7) can be used to control the reference point to move the cutter in X and Y directions. The Z-axis ultrasonic-assisted vibration displacement can be controlled by Equation (8), so that the tool can vibrate with high frequency along the axial direction in the process of axial feeding. Since the model applies a fixed constraint on the outer wall of the workpiece to limit the movement of the workpiece, the bearing reaction on the outer wall of the workpiece is equivalent to the three-dimensional force data measured by the dynamometer in the actual cutting experiment.

*x_t_* = *A*_1_ cos(*ω*_1_ + *φ*_1_) (6)

*y_t_* = *A*_1_ sin(*ω*_1_ + *φ*_2_) (7)

v_1_ = A_0_ + A_2_ cos(ω_1_t + *φ*_3_) (8)

where *x* is the displacement of the cutter along the X axis, *y* is the displacement of the cutter along the Y axis, *v*_1_ is the velocity of the cutter along the Z axis, *A*_1_ is the amplitude of the displacement periodic function, and the value is the same as the eccentricity. *A*_0_ is the axial feed velocity, *ω*_1_ is the circular frequency of the displacement function, *A*_2_ is the amplitude of the change of the ultrasonic assisted vibration velocity, ω_2_ is the circular frequency of the ultrasonic vibration, and *φ*_1_, *φ*_2_ and *φ*_3_ are the phase angles.

## 3. CFRP Ultrasonic-Assisted Helical Milling Cutter Performance Comparison Experiment

In order to compare the cutting performances of the two cutters, a comparative experiment between the flat-bottomed reverse milling cutter and the gradual-removal milling cutter was designed for milling holes under the condition of axial ultrasonic assistance. The VDL-1000E high-speed milling machining center was used for the lathe and the USBT40ER32 inductive rotating ultrasonic vibration handle was used for the ultrasonic auxiliary machining equipment. The experimental material is unidirectional CFRP plate with the dimension of 200 mm × 110 mm × 5 mm.

**Figure 5:**
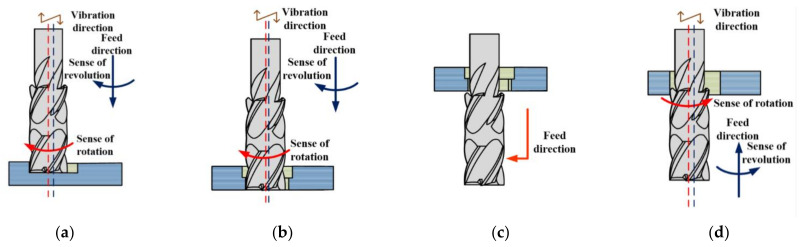
Bi-direction helical milling process strategy. (**a**) Large eccentricity helical milling; (**b**) small eccentricity helical milling; (**c**) adjust cutter position; (**d**) reverse helical milling.

The bi-direction helical milling process used in the experiment is shown in Figure 5, which consists of four stages: (a) Mill CFRP workpiece with 2 mm eccentricity and 4 mm feed depth. (b) Reduce the eccentricity to 1.2 mm and continue the helical milling to form a through hole. (c) Continue feeding the cutter so that the reverse cutting edge can probe the outlet of the hole and adjust the eccentricity to 2 mm again. (d) Reverse helical milling with the reverse cutting area which removes the cutting allowance left by the forward spiral milling. The direction of cutter rotation needs to be changed according to the structure of the cutter.

**Figure 6:**
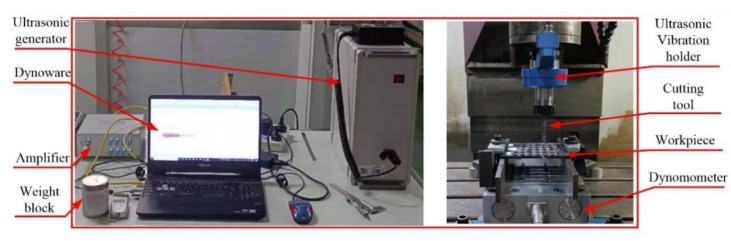
Experimental devices.

Before the formal experiment, a series of trial cutting tests based on the cutting parameters in the reference to optimize the experimental cutting parameters were conducted. Considering the surface quality and hole-making efficiency, the cutting parameters were optimized as follows: the rotational speed was 3600 r/min, the helical pitch was 0.25 mm, the axial feed rate was 7.5 mm/min, the axial feed per tooth was about 0.5 μm/z, and the vibration frequency was 22 kHz. The milling force was collected by Kistler 9139AA three-direction piezoelectric dynamometer. When the reverse cutting depth reached 30 mm, 60 mm and 90 mm, the industrial camera was used to collect the wear image of the cutting edge of the milling cutter. After the cutter was blunted, the cutter was unloaded, and the SU3500 scanning electron microscope was used to detect the flank wear morphology and microstructure of the reverse cutting edge of different cutters. The experimental devices are shown in Figure 6.

## 4. Results and Discussion

### 4.1. Influence of Cutter Structure on Cutting Force

**Figure 7:**
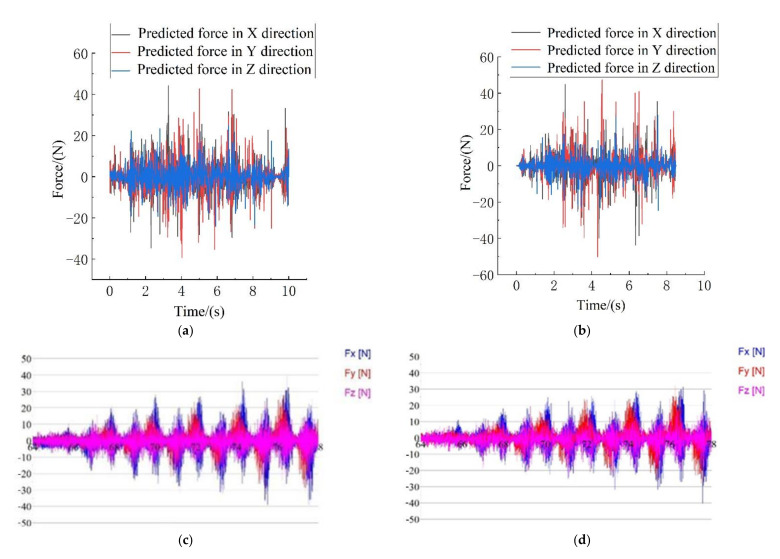
Comparison between experimental results and simulation results. (**a**) Simulation force of the gradual-removal reverse edge milling cutter; (**b**) simulation force of the flat-bottomed reverse edge milling cutter; (**c**) experimental force of the gradual-removal reverse edge milling cutter; (**d**) experimental force of the flat-bottomed reverse edge milling cutter.

**Figure 8:**
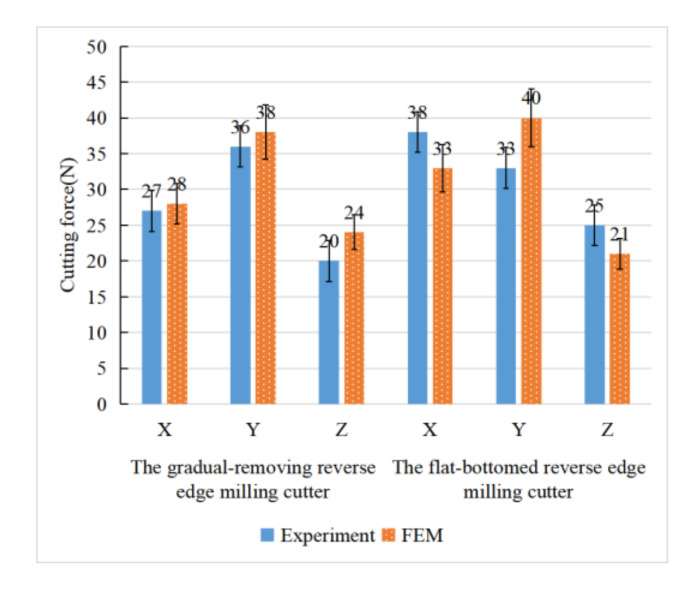
Comparison of cutting force between simulation model and cutting experiment.

In this paper, the finite element simulation model was established to predict the cutting force of different cutting cutters in reverse helical milling of CFRP exit materials, and the prediction results of the cutting force were verified by experiments. The simulation results of the cutting force are shown in Figure 7. When the cutting force fluctuation amplitude was in the stable stage, the average of the simulation and actual cutting peak forces were counted. The error of the cutting force was less than 25%, the statistical results are shown in Figure 8. The size of the error may be affected by the cutting heat, the actual mechanical properties of the material and the measurement error. In general, the cutting force error is within a reasonable range, which can be applied to the comparison of the cutting performance of the cutter structure and the optimization design of the cutter structure. Since the tool wear is light and the advantage of the tool structure is not obvious, there is no significant difference between the simulation results and the experimental data in the cutting force of the two milling cutters.

**Figure 9:**
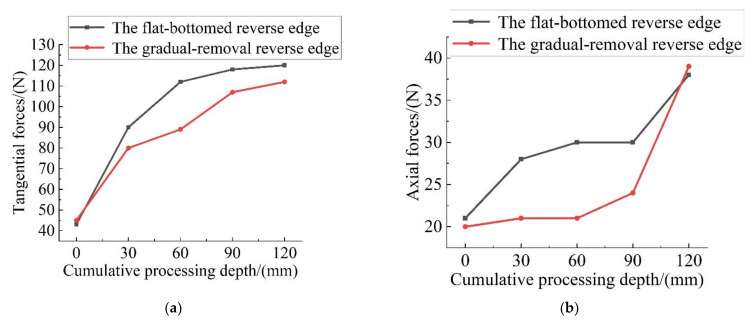
The variation curve of cutting force with cumulative cutting depth. (**a**) Tangential forces; (**b**) axial forces.

In order to compare the change of the cutting force after wear of different cutting cutters, the amplitude of the Z-direction cutting force fluctuation value in the cutting stability stage was counted, and the average value of the cutting force amplitude was calculated. The value was used as an evaluation index to measure the Z-direction cutting force in the machining process. The cutting forces in the X, Y two dimensions alone do not have a clear physical meaning. In order to study the change of the cutting force in the XY plane under cutter wear condition, the amplitude of the X, Y two-dimensional cutting force in the fluctuation stability stage should be respectively averaged, and the root mean square of these two values Fc should be found, which could be utilized as the tangential force reference index. The variation curves of the cutting force with the increase of cumulative cutting depth are shown in Figure 9.

The cutting force direction of the circumferential edge is perpendicular to the tangential direction of the spiral edge line, so the change of axial force is mainly affected by the axial component of the circumferential edge cutting force, and the change of tangential force reflects the overall wear degree of the tool. With the cutting progress, the tangential and axial cutting force of the flat-bottomed reverse edge milling cutter and gradual-removal reverse edge milling cutter showed a certain increase; when the cumulative machining depth was 30 mm to 90 mm, it entered the normal wear stage. Compared with the flat bottom reverse edge milling cutter, the axial cutting force produced by the gradual cutting reverse edge milling cutter was only 1/2 to 2/3 of the former. This is because the cutting task of the circumferential edge is reduced and the tool wear is reduced. When the cumulative machining depth reaches 120 mm, the tool edge produces severe wear, the tool edge becomes blunt, and the axial force of the two milling cutters increases significantly, and the numerical value is similar again.

### 4.2. Influence of Cutter Structure on Cutter Wear

The support reaction can only represent the overall cutting force of the cutter. In order to further study the influence of the cutter structure on the cutting force and cutter wear, it is necessary to analyze the output results of field variables. In this simulation model, the cutter structure was set as a rigid body, and among the results of the researchable variables of the rigid body, the dynamic change of the positive pressure on the nodes could most reflect the change trend of the cutting force. A typical moment was selected to clarify the influence of the cutter structure on the cutting force distribution combined with the wear condition of the cutter.

**Figure 10:**
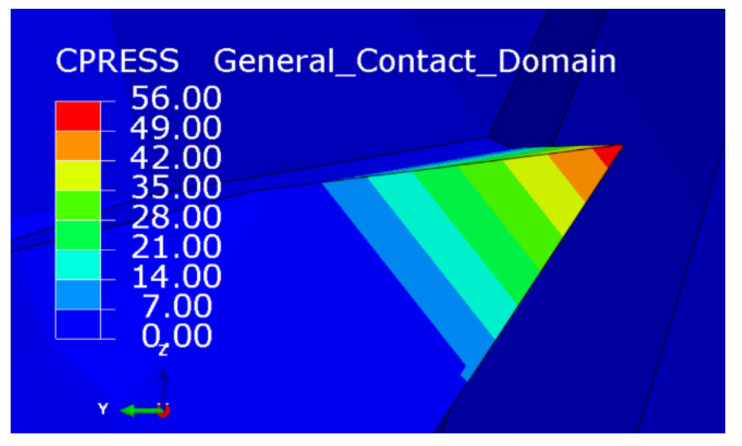
The cutting force distribution of the flat-bottomed reverse edge.

**Figure 11:**
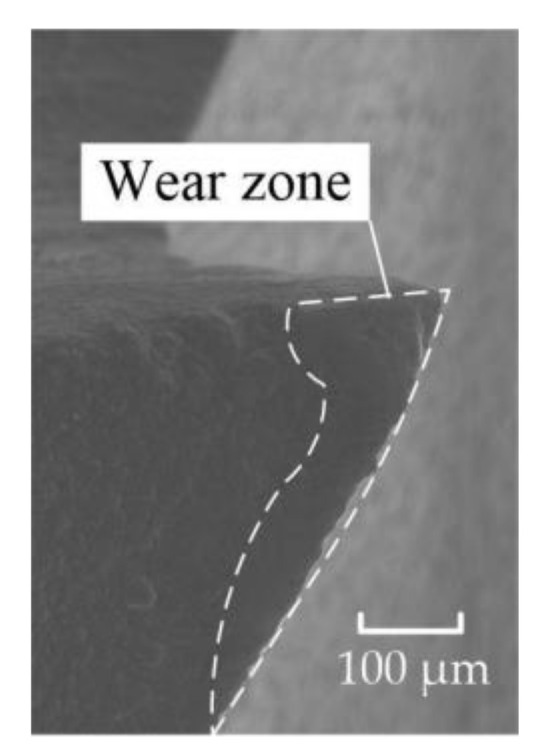
The wear zone distribution of the flat-bottomed reverse edge.

In the process of flat-bottomed reverse cutting, the contact area between the cutter and the material was small, and the material removal task was jointly undertaken by the end edge and the peripheral edge, which made the pressure concentration point of the cutter located at the junction of the peripheral edge and the end edge, as shown in Figure 10. The flank face wear morphology of the flat-bottomed reverse edge milling cutter in reverse cutting is shown in Figure 11. Since the fiber is a typical material with rebound characteristics and high strength, the cutter was susceptible to abrasive wear due to the influence of hard particles in the fiber. Therefore, obvious wear bands can be observed on the flank face of the cutter.

**Figure 12:**
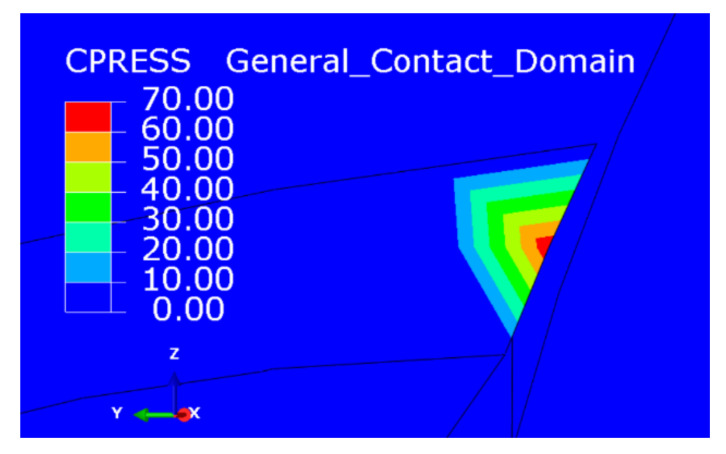
The cutting force distribution of the gradual-removal reverse edge.

**Figure 13:**
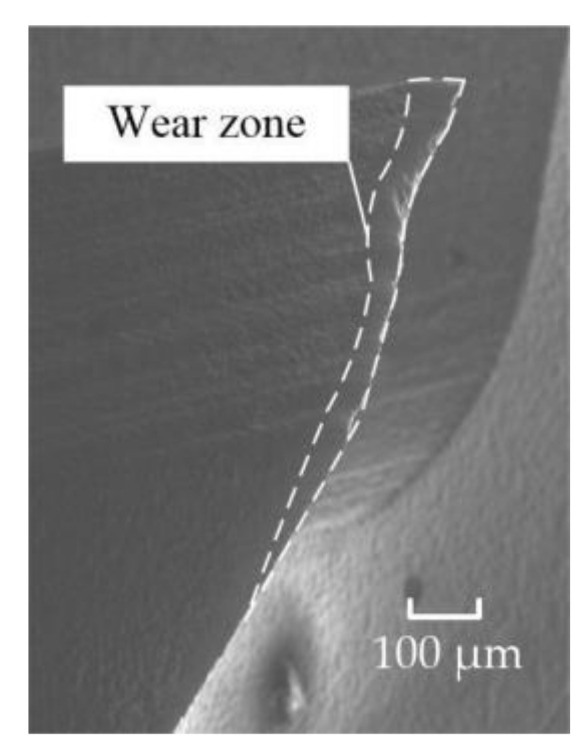
The wear zone distribution of the gradual-removal reverse edge.

The cutting force of the gradual-removal reverse edge milling cutter was mainly concentrated in the cutter tip when it cuts into the material. With the continuous feed of the cutter, the pressure concentration point gradually shifted from radial to lateral. In the stable cutting stage, the positive pressure distribution of the gradual-removal reverse edge milling cutter was shown in Figure 12. It could be inferred from the cloud picture of the force situation that the cutting force of the flat-bottomed milling cutter was concentrated at the tool nose, while the cutting task is transferred to the end edge during the cutting process of the cutter with a gradual-removal reverse edge. Although the increase in the end edge cutting task would lead to faster wear, the end edge did not participate in the machining of the hole wall, so the influence of the end edge wear on the quality of the hole wall was also smaller than that of the peripheral edge. Figure 13 shows the wear morphology of the back flank of the reverse cutting zone on the gradual-removal reverse edge milling cutter. It could be observed that the wear band of the flank face wear started from the tip and extended along the end edge to the peripheral edge. The wear zone at the end edge was wider, and an obvious notch depression could be seen at the edge, where the diamond coating had almost completely fallen off. It could be seen that the wear degree of the end edge was far more serious than that of the peripheral edge. Under the assistance of axial ultrasonic vibration, the end edge contacted with the part to be machined intermittently, and its life could also be guaranteed. The gradual-removal edge structure could well protect the peripheral edge, reduce the friction of the peripheral edge, and extend the overall service life of the cutter.

**Figure 14:**
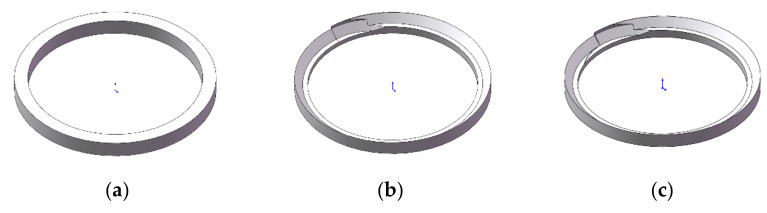
The establishment process of chip simulation model. (**a**) Part of the materials to be processed; (**b**) remaining materials after one circle of revolution; (**c**) remaining materials after a circle of rotation.

In order to further study the cutting ratio of the peripheral edge and the end edge of different cutter structures, it is accessible to simulate the undeformed chip of each tooth based on specific cutting parameters in the experiment. The process of model establishment is shown in the Figure 14. The specific implementation method contains 4 steps: First, draw the shape of the material that will be removed in the reverse cutting process in SolidWorks 2017 (Dassault Systemes, Concord, MA, USA). Second, according to the cutter structure and cutting parameters, the geometric shape of the remaining materials after a circle of milling cutter revolution should be plotted by using the rotary cutting instruction. Then, according to the same method, draw the remaining material after a circle of rotation of the cutter on the basis of the previous step. Finally, the solid models obtained in the second and third steps should be used to calculate the difference set by the Boolean operation, and the approximate undeformed chip shape of each tooth can be obtained.

**Figure 15:**
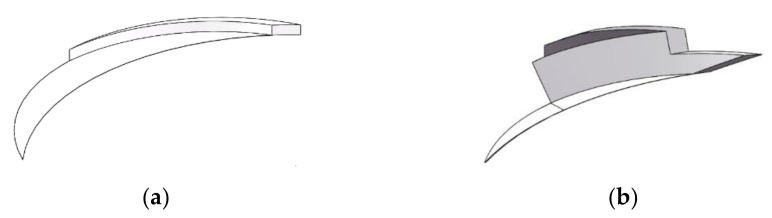
Chip simulation. (**a**) Chips formed by the flat-bottomed reverse edge cutting cutter; (**b**) chips formed by the gradual-removal reverse edge cutting cutter.

Due to the structural characteristics of carbon fiber composite materials, the chip shape powdered, and the chip diameter is generally tens to hundreds of micrometers. Therefore, the shape of the undeformed chip per tooth can only be used to reflect the influence of the cutter structure on the material cutting task allocation, and cannot reflect the actual shape of the chip. The undeformed chip simulation per tooth of the actual flat-bottomed reverse cutting edge and the gradual-removal reverse cutting edge are shown in Figure 15a,b, respectively.

The figure shows that the shape of the reverse cutting material is crescent, and extends from the hole wall to the hole center. In the process of gradual-removal reverse edge cutting, the peripheral edge of the cutter removes part of the material. When the cutter turns to the same position in XOY, the end edge of the cutter removes less part of the material, which means the cutting task of the peripheral edge of the cutter, in the next turn, to the same position, is effectively reduced. The end edge is responsible for removing materials and expanding holes, while the peripheral edge is only responsible for removing materials near the hole wall.

**Figure 16:**
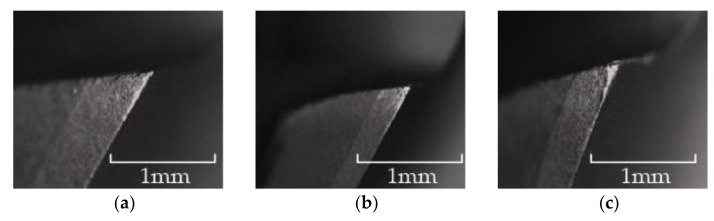
Wear morphology of flat-bottomed reverse edges. (**a**) Cumulative depth of 30 mm; (**b**) cumulative depth of 60 mm; (**c**) cumulative depth of 90 mm.

As shown in Figure 16, in the wear experiment, when the cumulative reverse machining depth of the flat-bottomed reverse edge milling cutter was 30 mm, the obvious bright wear band could be observed at the tool nose of the end edge and the peripheral edge of the reverse cutting zone of the cutter, and the cutter began to wear obviously at this time. When the cumulative reverse cutting depth of the flat-bottomed reverse edge milling cutter was 60 mm, the wear band of the cutter extended downward from the tool nose of the cutter along the peripheral edge. When the cumulative reverse cutting depth of the flat-bottomed reverse edge milling cutter was 90 mm, the wear zone of the cutter edge extended from the cutting edge to the flank face, forming a triangular wear area, and the cutter wear was serious.

**Figure 17:**
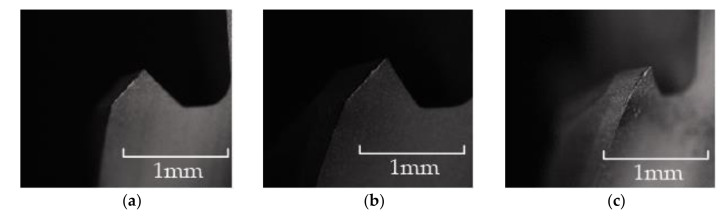
Wear morphology of gradual-removal reverse edges. (**a**) Cumulative depth of 30 mm; (**b**) cumulative depth of 60 mm; (**c**) cumulative depth of 90 mm.

As shown in Figure 17, when the cumulative reverse cutting depth of the involute reverse milling cutter was 30 mm, the end edge of the reverse cutting cutter produced bright spots. When the cumulative machining depth was 60 mm, the wear bright spots of the end edge connected to form a complete wear band, and the peripheral edge had no obvious wear. When the cumulative depth was 90 mm, the wear band extended to the peripheral edge, and the intersection of the end edge and the peripheral edge produced concentrated wear, and extended to the back cutter surface; the cutter was blunt, and the experiment was terminated.

**Figure 18:**
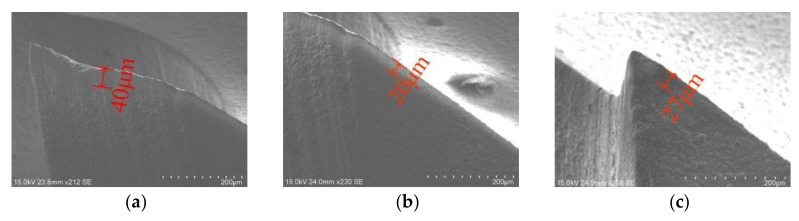
Comparison of flank wear width. (**a**) The gradual removal reverse end edge; (**b**) the gradual-removal reverse peripheral edge; (**c**) the flat-bottomed reverse peripheral edge.

Figure 18 compared the width of the cutter wear band after the reverse cutting of the two milling cutters. It could be seen that the wear band of the gradual-removal reverse end edge was about 40 microns, while that of the peripheral edge was about 20 microns. From the end edge to the peripheral edge, the width of the cutter wear band decreased gradually. In contrast, the wear band width of the peripheral edge forming the flat-bottomed reverse edge milling cutter was wider, thus, the peripheral edge of the gradual-removal reverse edge milling cutter was better protected.

In summary, with the increase in the number of holes, the wear of the reverse cutting area of the flat-bottomed reverse edge milling cutter was relatively serious, and the wear area was concentrated at the junction of the end edge and the peripheral edge. The wear degree of the peripheral edge from the gradual-removal reverse edge milling cutter was lighter, because the end edge undertook most of the cutting tasks, better protected the peripheral edge and reduced the friction between the peripheral edge and the workpiece. Without changing the processing technology, the cutting amount of the edge was reduced, the finishing of the hole wall position material was realized, and the life of the cutter edge was prolonged.

### 4.3. Influence of Cutter Structure on Quality of Hole Outlet

**Figure 19:**
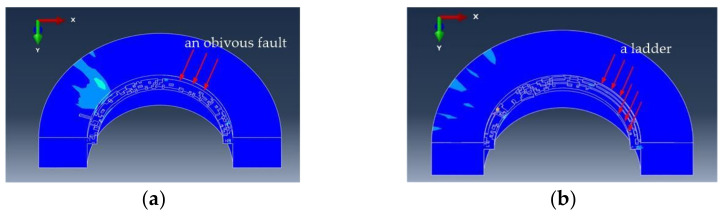
Comparison of two workpieces on 2 s time. (**a**) Residual material processed by the flat-bottomed reverse edge cutter; (**b**) residual material processed by the gradual-removal reverse edge cutter.

Figure 19a,b, respectively, show the simulation state of milling the CFRP outlet at the time of 2 s by a flat-bottomed reverse edge milling cutter and gradual-removal reverse edge milling cutter. It could be observed that there was an obvious fault between the unprocessed material and the boundary layer of the flat-bottom reverse edge milling cutter. This is because during the straight-edge machining process, with the continuous feed of the cutter, the material near the hole wall was removed due to the cutting of the edge, and the distance between the boundary layer and the machined plane was equal to the depth of the axial feed. The material removal methods were different when the gradual-removal reverse edge milling cutter was cutting. The material to be processed in the hole presented a ladder shape. This is because at the beginning of reverse helical milling, the tool nose of the cutting edge contacted with the workpiece first. With the continuous feed of the cutter, the outer end of the reverse edge gradually participated in the cutting, which made the ladder move down and the aperture gradually increased. Therefore, the cutting process was gentler and stabler, realizing the finish machining of hole wall.

**Figure 20:**
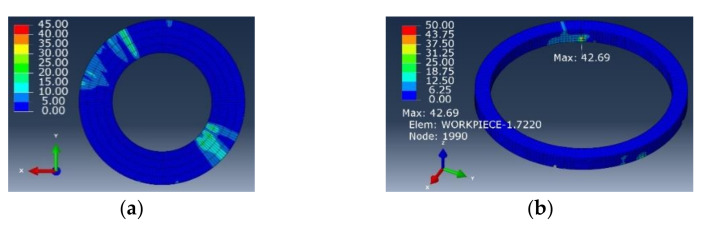
**Figure 20.** Simulation results of machining holes with the flat-bottomed reverse edge milling cutter. (**a**) Boundary layer; (**b**) hole wall unit at exit.

**Figure 21:**
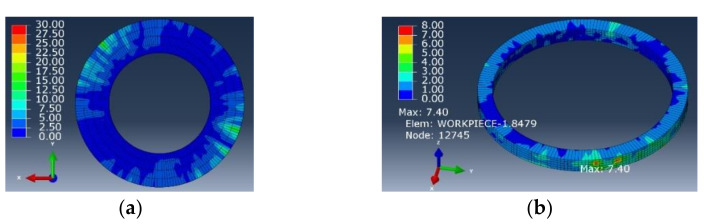
**Figure 21.** Simulation results of machining holes with the gradual-removal reverse edge milling cutter. (**a**) Boundary layer; (**b**) hole wall unit at exit.

Figure 20 and Figure 21 show the stress distribution nephogram of the interface layer at the exit of the workpiece processed by the flat-bottomed edge cutter and the gradual-removal edge cutter. The surface quality of the interface layer at the hole wall and the exit was good, and the stress of the hole wall changed with the change of the fiber angle. In contrast, the residual stress of the workpiece after gradual cutting was smaller. Through the stress distribution nephogram of the unit workpiece near the hole wall, it could be observed that the maximum ultimate stress of the hole wall formed by the flat-bottomed edge machining could reach 42.69 MPa, and the maximum stress of the hole wall formed by the gradual-removal edge machining is only 7.40 MPa, which was about 1/6 of the former. This indicates that in the actual processing, compared with the flat-bottomed reverse edge milling cutter, the quality of the hole wall processed by the gradual cutting reverse edge milling cutter was better, and the damage was significantly inhibited.

**Figure 22:**
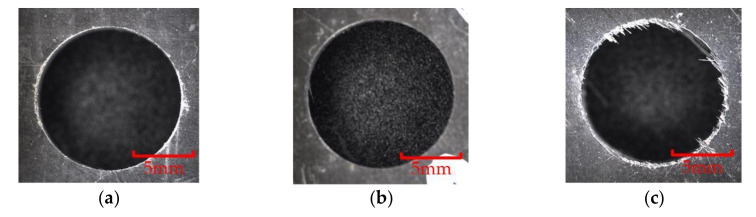
Hole quality at outlet of the gradual-removal reverse milling cutter. (**a**) Cumulative depth of 30 mm; (**b**) cumulative depth of 60 mm; (**c**) cumulative depth of 90 mm.

**Figure 23:**
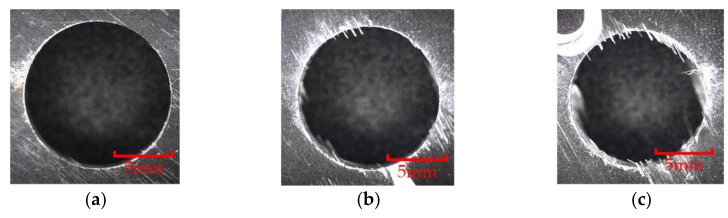
Hole quality at outlet of the flat-bottomed reverse milling cutter. (**a**) Cumulative depth of 30 mm; (**b**) cumulative depth of 60 mm; (**c**) cumulative depth of 90 mm.

When the cutter was not worn, due to the sharp cutting edge, there was little damage at the outlet of the hole. However, when the edge was blunted, the cutting performance of the cutter decreased, and the damage at the outlet of the hole was easy to occur. The difference in the quality of the outlet of the holes processed by different cutters was more obvious. Figure 22 and Figure 23 show the quality comparison at the outlet of the holes when the cumulative machining depths of the two milling cutters were 30 mm, 60 mm and 90 mm. When the cumulative machining depth was 30 mm, the surface quality at the outlet of the two milling cutters were both good; when the cumulative machining depth reached 60 mm, the quality at the outlet of the hole were significantly different. The quality at the outlet of the hole machined by the gradual-removal reverse edge milling cutter remained at a high level, and there was almost no burr. However, due to the wear at the interface between the peripheral edge and the end edge, a small amount of burr could be observed at the joint surface of the forward and reverse machining, and obvious burrs and tears could be observed at the boundary layer at the outlet processed by the flat-bottom reverse edge milling cutter. When the cumulative machining depth reached 90 mm, the quality at the outlet of the machining holes of the two kinds of cutters decreased significantly. The fracture mode of fiber changes from shear failure to extrusion failure. There were fewer burrs at the fiber angle of about 0° than burrs at the position of the fiber angle of about 90°. Due to the large area of fiber concession, a large number of burrs could be observed, which were consistent with the distribution of the maximum stress in the simulation results. At this time, the hole quality was difficult to meet the working requirements.

### 4.4. Discussion

Compared with the flat-bottomed reverse edge milling cutter, the gradual-removal reverse edge milling cutter designed in this paper has obvious advantages in the ultrasonic assisted bi-direction helical milling process. When the reverse cumulative cutting depth of the gradual-removal reverse edge is about 30 mm to 90 mm, the peripheral edges are well protected, so the axial force, which is affected by the sharpness of the peripheral edge, is only 1/2 to 2/3 of the flat-bottomed reverse edge milling cutter, the width of the wear band is also smaller, and the burr damage at the exit is also significantly suppressed.

In order to clarify the reason why the gradual-removal reverse edge milling cutter has obvious advantages in tool life, the Abaqus cutting simulation models of two milling cutters under ultrasonic assisted conditions were established. By comparing the actual value of the cutting force with the simulation value, it is known that the maximum error of the two cutting forces is 25%, which indicates that the simulation model is accurate enough. By observing the force on the cutting edge of the cutting tool during the cutting process, and combining with the SolidWorks simulation model of an undeformed chip, it can be seen that the cutting force of the gradual-removal reverse edge milling cutter is concentrated at the reverse end edge, and the cutting task of the flat-bottomed reverse edge milling cutter is more borne by the peripheral edge.

## 5. Conclusions

In this paper, under the condition of ultrasonic vibration assistance, the simulation and experimental study of gradual-removal reverse edge milling CFRP was carried out, and the finite element models of three-dimensional helical milling CFRP with the gradual-removal reverse edge milling cutter and the flat-bottomed reverse edge milling cutter were established. The cutting performance and cutter life of two milling cutters were analyzed, and the following conclusions were drawn:

(1) Through the ultrasonic-assisted helical milling of CFRP finite element model, the cutting force and the stress distribution of the hole wall processed by the two helical milling cutters were obtained. The material removal behavior in the dynamic cutting process was simulated and predicted, and the mechanism of the gradual-removal reverse edge milling cutter to improve the cutter life and machining quality were intuitively revealed.
(2) The wear of the edge of the flat-bottomed reverse edge milling cutter is relatively serious, and the wear of the edge of the gradual-removal reverse edge milling cutter is relatively light. The width of the wear band of the edge is only 20 μm, which is still relatively sharp. In the normal wear stage, the axial force of the gradual-removal reverse edge milling cutter increases slowly, which is only about 1/2 to 2/3 of the flat-bottomed reverse edge milling cutter.
(3) The cutting ratio of the end edge and the peripheral edge was obtained by simulating the undeformed chip of each tooth. The end edge of the gradual-removal reverse edge milling cutter removed some materials that should be removed by the peripheral edge, so that the proportion of the peripheral edge participating in the cutting was significantly reduced, and the cutter life and the quality of the hole were effectively improved.
(4) According to the cutting simulation results, the maximum stress in the boundary layer of the machining hole processed by the gradual-removal reverse edge milling cutter was about 1/6 of that of the flat-bottom reverse edge milling cutter. Due to the slow wear of the peripheral edge of the gradual-removal reverse edge milling cutter, the material removal form of the gradual-removal reverse edge milling cutter is still dominated by shear failure at the reverse cutting depth of about 60 mm, and the machining defects at the outlet of the hole are effectively suppressed.

## Figures and Tables

**Table 1:** Summary of main information of referenced Researcher.

	Main Research Objects	Diameter (mm)	Rotation Speed	Pitch (mm)	Material
Sadek [7]	Comparison between conventional drilling and helical milling	9	6000~16,000 r/min	1 and 3	CFRP
Wang [8]	Comparison between conventional drilling and helical milling	5	3000 r/min	0.5	CFRP and Ti alloy
Amini [9]	Comparison between helical milling with different cutting parameters	10	40, 70, 100 m/min	0.15	CFRP
Brinksmeier [10]	Cutting ratio between peripheral edge and end edge				
Han [11]	Comparison between tools of different shapes and coats	10	3000 r/min	1	CFRP
Chen [12]	Comparison between specialized milling cutter and flat end milling cutter	15	3000 r/min	0.2	CFRP and Ti alloy
Yang [13]	Forward and reverse helical milling	10	5000 r/min	0.33	CFRP
Ishida [14]	Comparison between ultrasonic assisted and low temperature cooling assisted machining methods	5	8000 r/min	1	CFRP
Chen [15]	Comparison between ultrasonic assisted and traditional helical milling	8	4500 r/min	0.2	CFRP
Liu [16]	Comparison of different vibration forms	10	6000 r/min	0.15	CFRP

**Table 2:** Simulation performance parameters of T700.

Parameters	Values
Density (g/cm^3^)	*ρ =* 1.81
Elastic modulus (GPa)	*E*_1_ = 233, *E*_2_ = *E*_3_ = 15
Poisson ratio	*v*_12=_*v*_13_ = 0.21, *v*_23_ = 0.35
Shear modulus (GPa)	*G*_12_ = *G*_13_ = 24, *G*_23_ = 5.03
Tensile strength (MPa)	*σ^T^*_11_ = 1830, *σ^T^*_22_ = 37.7
Compressive strength (MPa)	*σ^C^*_11_ = 872, *σ^C^*_22_ = 137
Shearing strength (MPa)	*S*_12_ = *S*_13_ = *S*_23_ = 69.7

**Table 3:** Simulation Performance Parameters of cutters.

Parameters	Values
Density (g/cm^3^)	*ρ =* 14.6
Elastic modulus (GPa)	*E*_T_ = 64
Poisson ratio	*v*_t_= 0.22

## Data Availability

Not applicable.
